# RevUP: an online scoring system for regulatory variants implicated in rare diseases

**DOI:** 10.1093/bioinformatics/btac157

**Published:** 2022-03-15

**Authors:** Solenne Correard, Brittany Hewitson, Robin van der Lee, Wyeth W Wasserman

**Affiliations:** Department of Medical Genetics, Centre for Molecular Medicine and Therapeutics, BC Children's Hospital Research Institute, University of British Columbia, Vancouver, BC V5Z 4H4, Canada; Department of Medical Genetics, Centre for Molecular Medicine and Therapeutics, BC Children's Hospital Research Institute, University of British Columbia, Vancouver, BC V5Z 4H4, Canada; Department of Medical Genetics, Centre for Molecular Medicine and Therapeutics, BC Children's Hospital Research Institute, University of British Columbia, Vancouver, BC V5Z 4H4, Canada; Department of Medical Genetics, Centre for Molecular Medicine and Therapeutics, BC Children's Hospital Research Institute, University of British Columbia, Vancouver, BC V5Z 4H4, Canada

## Abstract

**Summary:**

To address the difficulty in assessing the implication of regulatory variants in diseases, a scoring scheme previously published allows the calculation of the Regulatory Variant Evidence score (RVE-score). The score represents the accumulated evidence for a causative role of a regulatory variant in a disease. Regulatory Evidence for Variants Underlying Phenotypes was built to calculate the RVE-score of regulatory variants, based on the 24 criteria, with a hybrid approach combining information retrieved from public databases and user input.

**Availability and implementation:**

RevUP is freely available at http://www.revup-classifier.ca. The source code is available at https://github.com/wassermanlab/revup.

**Supplementary information:**

Supplementary data are available at *Bioinformatics* online.

## 1 Introduction

To date, several DNA variant classifiers are available and most are aligned to the American College of Medical Genetics and Genomics (ACMG) consensus recommendations (Richards *et al.*, 2015). Recently, recommendations were proposed for changes to the ACMG guidelines for clinical interpretation of variants to include variants found in non-coding regions of the genome, showing the relevance of such variants for genetic conditions (Ellingford *et al.*, 2021; Turner and Eichler, 2019).

With the growing use of clinical whole-genome sequencing, there is a need for extension of the classification systems suitable for regulatory sequence variants. Diverse regulatory variant prediction methods have been developed, such as PRVCS (Li *et al.*, 2016) or GREEN-VARAN (Giacopuzzi *et al.*, 2020), have begun to aggregate predictions and prioritize variants expected to disrupt gene regulation. Such bioinformatics tools generally draw upon genomics databases for information, but often the strongest evidence is found in gene-specific publications. Furthermore, the focus on regulatory dysfunction does not necessarily relate to pathogenic impact, which is critical for clinical classification. In 2020, a review of 46 regulatory disease variants reported to disrupt the expression of 40 transcription factor genes generated a semiquantitative classification scheme that incorporates both functional and clinical evidence (van der Lee *et al.*, 2020). The scheme allows the calculation of the Regulatory Variant Evidence score (RVE-score), based on 24 criteria, which summarizes the accumulated evidence for a regulatory variant to have a causative role in a rare disease.

Regulatory Evidence for Variants Underlying Phenotypes (RevUP, http://www.revup-classifier.ca), a web-based classifier, was built to calculate the RVE-score of regulatory variants, based on the 24 criteria presented in van der Lee *et al.* (2020). Some previously released classifiers (not regulation focused) rely on user input only (Kleinberger *et al.*, 2016), while others rely uniquely on information available in public databases (Li and Wang, 2017), RevUP relies on both. The result page is a user-friendly display, downloadable and suitable for inclusion in a scientific report, together with a FAQ on the use of RevUP.

## 2 Materials and methods

### 2.1 Architecture and hosting

RevUP is a web application composed of a front end written in React, and a backend framework written in Flask. It is hosted on Amazon Web Services using a t2.medium elastic compute (EC2) instance with Nginx being used as the web server to route traffic. All code for RevUP is available on GitHub https://github.com/wassermanlab/revup.

### 2.2 Scoring scheme

The scoring scheme presented in van der Lee *et al.* (2020) is composed of a clinical component ‘Is there a causal link between genotype and phenotype?’ and a functional component ‘Does the variant have a damaging effect on the gene?’. Each evidence is given a score of 0 or 1 (0: The evidence does not apply to the variant or it is unknown; 1: The evidence applies to the variant, pondered based on the evidence weight). From the evidence, three scores are calculated: (i) the C-score, reflecting the clinical evidence available; (ii) the F-score, reflecting the functional evidence available and (iii) the RVE-score which is the accumulated evidence for a causative role of a regulatory variant in a disease. For some of the evidence, the information can be retrieved from public databases while other evidence requires user input (ex: C3.1, ‘Variant shows familial segregation with the disease’).

### 2.3 User input on the variant and target gene (Step 1) and databases queried by the webserver

For RevUP to query external databases, the user must input the variant details and the suspected target gene. Six external resources are then queried: PhyloP (Cooper *et al.*, 2005), PhastCons (Siepel *et al*., 2005), gnomAD (Karczewski *et al*., 2020), CADD (Rentzsch *et al*., 2019), ReMap (Chèneby *et al*., 2020) and ENCODE (Davis *et al*., 2018). The details on the versions used and the information retrieved can be found in Supplementary Table S1.

### 2.4 User input for additional evidence (Step 2)

Some evidence cannot be found online. Others can be found using public databases but are too complicated to query automatically as they require interpretation by a human expert (ex: C2.1, ‘Suspected target gene has been implicated in the same or a similar disease phenotype, or is otherwise relevant’). Therefore, the user will have the ability to answer ‘Yes’ or ‘No’ to these questions in order for the tool to calculate the score.

### 2.5 Results modification and comments (Step 3)

Users may have additional evidence that was not available in the queried public databases, therefore, the user can modify the score for each evidence level as well as add comments. Comments were created to allow citations to relevant publications, or specific figures within publications (i.e. free text). It is not advised to change the scores unless the user has strong evidence to justify it.

## 3 Results

**Fig. 1.**
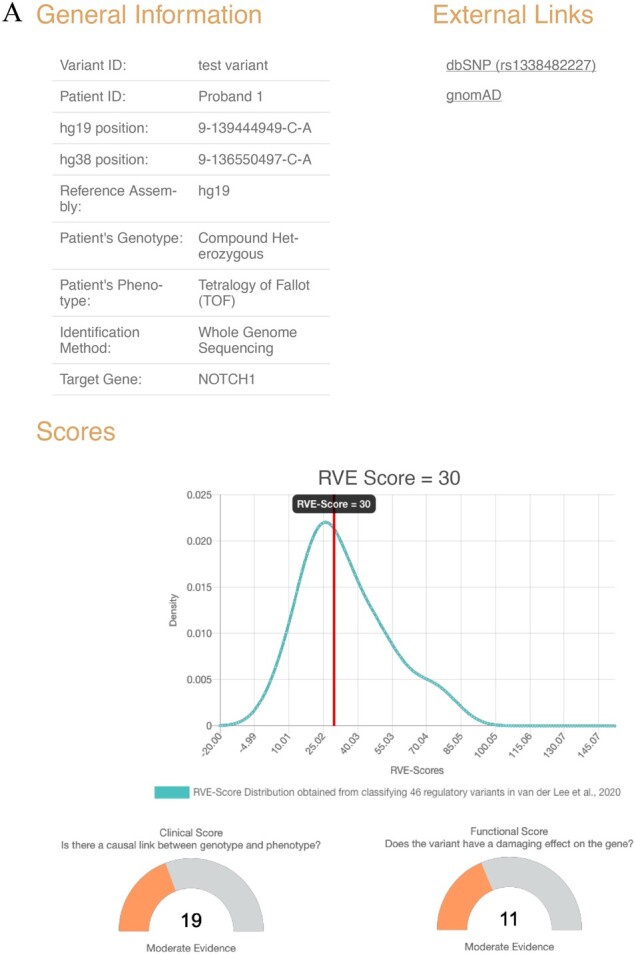
RevUP report obtained for the scoring of the non-coding variant in the NOTCH1 gene reported by Wang *et al*. (2020) in proband 1. (**A**) Summary information for the variant; (**B**) clinical and functional information compiled from user input and external databases used to generate the RevUP score

Based on the user input and on the values retrieved from the external databases, RevUP calculates the C-score, the F-score and the RVE-score for the submitted variant. The result page is composed of two parts. The top portion (Fig. 1A) presents the information concerning the variant, the RVE-score and the strength of this score relative to the distribution of a curated collection of 46 regulatory variants (van der Lee *et al.*, 2020). This can indicate to the user if the score is high or low compared to published variants. Then, the C- and F-scores are displayed separately for the user to assess the strength and weakness of their analysis. The bottom portion (Fig. 1B) indicates, for each evidence level, the score, the information outputted from public databases and the comments added by the user (if any). The results are downloadable in PDF or png formats to present as-is or in modified form.

An example is shown in Figure 1, based on a recently published variant located in a regulatory region upstream of *NOTCH1* and implicated in the tetralogy of Fallot (Wang *et al.*, 2020). We input the variant characteristics and the information found in the paper in RevUP. The tool allowed for a quick scoring of the variant, and the creation of the displayed figure.

## 4 Conclusions and outlook

RevUP is a strong addition to the available variant classifier tools, as it will allow users to assess properties specific to regulatory variants. This scoring system does not conflict with the ACMG classification guidelines; rather it can be used as additional information when studying regulatory variants. The tool will save time for the user as it is able to query databases rapidly.

## Supplementary Material

btac157_Supplementary_Data
