# Supplementary material for: RevUP: an online scoring system for regulatory variants implicated in rare diseases
Source: Bioinformatics. 2022 Mar 15;38(9):2664–6. doi: 10.1093/bioinformatics/btac157 (PMC9048665; doi:10.1093/bioinformatics/btac157)
Supplement: btac157_Supplementary_Data [file btac157_supplementary_data.pdf]

Supplementary Figure

Evidence level : Evidence for pathogenicity	Source of information	Information extracted	Scoring	Query
C1.1: Variant position is evolutionarily conserved	PhyloP (Cooper et al., 2005)	PhyloP score	PhyloP > 1.5 or	UCSC REST API; track: phyloP100way; hg38
	PhastCons (Siepel et al., 2005)	PhastCons score	PhastCons > 0.5	UCSC REST API; track: phastCons100way; hg38
C1.2 : Variant is rare in unaffected individuals in specific sets of controls or reference population databases	gnomAD v3.1.1 (Karczewski et al., 2020)	Allele count (AC) Allele Number (AN) Number of homozygotes	gnomAD AF < 0.05	gnomAD GraphQL; dataset: gnomad_r3; hg38
C2.3 : Variant is considered deleterious by computational prediction methods	CADD v1.6 (Rentzsch et al., 2019)	CADD phred score	CADD > 15	CADD REST API; v1.0; GRCh38
F1.1 : Variant position is implicated in TF binding based on experimental data	ReMap 2020 (Chèneby et al., 2020)	TF included in overlapping Cis-Regulatory Modules (CRMs)	If the variant position intersect with at least one CRM → 1; else → 0	Intersect with file downloaded from ReMap 2020, Homo Sapiens, CRMs, hg38 (1,7 million)
F1.2 : Variant localizes to a regulatory region based on genome annotations	ENCODE / SCREEN (Davis et al., 2018)	cCRE details : cCRE, description and name	If the variant position intersect with at least one cCRE → 1; else → 0	UCSC REST API; track: encodeCcreCombined; hg38
F1.3 : Regulatory region and target gene are directly linked based on annotation or experimental data		Method and cell type	if F1.2 = 0 → 0; else, if cCRE and potential target gene linked based on eQTL method → 1; else → 0	SCREEN GraphQL; GRCh38
F1.4 : Variant is statistically associated with expression levels of the target gene			if F1.2 = 0 → 0; else, if cCRE and potential target gene linked based on Hi-C or CHIA-PET method → 1; else → 0	

Supplementary Table 1. External public databases used to score seven pieces of evidence in RevUP and details of the scoring system for these evidence levels. Details on the evidence level description can be found on RevUP. cCRE: candidate cis-Regulatory Elements.
